# THE MEDIATING ROLE OF SOCIAL ISOLATION BETWEEN FALLS AND COGNITIVE FUNCTION AMONG OLDER ADULTS IN THE UNITED STATES

**DOI:** 10.1093/geroni/igae098.0362

**Published:** 2024-12-31

**Authors:** Wonkyung Jung, Janiece Taylor

**Affiliations:** Boston College, Baltimore, Maryland, United States; Johns Hopkins University, Johns Hopkins University, Maryland, United States

## Abstract

Social isolation and falls are significant concerns affecting the cognitive health of older adults. However, little is known about the interrelationships between social isolation, falls, and cognitive function among older adults in the United States (U.S.). This study aims to investigate the interrelationships between these factors and the mediating role of social isolation on the relationship between fall experiences in the past 12 months and cognitive function among older adults in the U.S. Utilizing Round 5 data from the National Health and Aging Trends Study (NHATS), we employed linear regression models using Hayes’s PROCESS macro with 5,000 bootstrapped mediation analyses in SPSS. Of the 947 participants, 60.8% were younger than 75 years, 38.6% were women, and most (77.1%) were White. Our findings revealed that social isolation did not mediate the relationship between falls and cognitive function. Additionally, we found no significant association between fall experiences and both social isolation and cognitive function. However, our analysis identified that socially isolated older adults were more likely to experience cognitive impairments (β =.84, P <.001). These results highlight the complex nature of the relationships among social isolation, falls, and cognitive function in older adults. Further research is warranted to explore potential mechanisms underlying the observed associations and to develop targeted interventions to address cognitive impairments among socially isolated individuals. Strategies targeting social connectedness may have promising implications for promoting cognitive health in older adults in the U.S.

